# Synthesis of acetamidosulfonamide derivatives with antioxidative and QSAR studies

**DOI:** 10.17179/excli2021-4590

**Published:** 2022-02-01

**Authors:** Apilak Worachartcheewan, Somchai Pisutjaroenpong, Ratchanok Pingaew, Supaluk Prachayasittikul, Suphakit Siriwong, Somsak Ruchirawat, Virapong Prachayasittikul

**Affiliations:** 1 Department of Community Medical Technology, Faculty of Medical Technology, Mahidol University, Bangkok 10700, Thailand; 2 Laboratory of Medicinal Chemistry, Chulabhorn Research Institute, Bangkok 10210, Thailand; 3 Department of Chemistry, Faculty of Science, Srinakharinwirot University, Bangkok 10110, Thailand; 4 Center of Data Mining and Biomedical Informatics, Faculty of Medical Technology, Mahidol University, Bangkok 10700, Thailand; 5 Program in Chemical Science, Chulabhorn Graduate Institute, Bangkok 10210, Thailand; 6 Center of Excellence on Environmental Health and Toxicology, Commission on Higher Education (CHE), Ministry of Education, Thailand; 7 Department of Clinical Microbiology and Applied Technology, Faculty of Medical Technology, Mahidol University, Bangkok 10700, Thailand

**Keywords:** sulfonamides, antioxidants, superoxide dismutase, QSAR, rational design

## Abstract

A series of sixteen acetamidosulfonamide derivatives (**1**-**16**) have been synthesized and investigated for their antioxidant (radical scavenging and superoxide dismutase (SOD)) and antimicrobial activities. Most compounds exhibited antioxidant activities in which compound **15** displayed the most potent radical scavenging and SOD activities. Quantitative structure-activity relationship (QSAR) has been studied using multiple linear regression. The constructed QSAR models displayed high correlation coefficient (*Q*^2^*_LOO-CV _*= 0.9708 and 0.8753 for RSA and SOD activities, respectively), but low root mean square error (*RMSE_LOO-CV _= *0.5105 and 1.3571 for RSA and SOD activities, respectively). The structure-activity relationship showed that an ethylene group connected to pyridine ring provided significant antioxidant activities. The QSAR models give insight into the rational designed of eighty new sulfonamides with various electron donating and withdrawing groups. The top five new designed sulfonamides with nitro group are potential antioxidants to be further developed for medicinal applications.

## Introduction

Free radical, a molecule having an unpaired electron, plays physiological signalings in the body such as cell growth, hormone synthesis and killing microorganisms in normal condition (Droge 2002[11]; Knight 2000[18]; Lobo et al., 2010[20]). However, excessive or uncontrolled free radical productions known as oxidative stress cause biological alterations and eventually chronic diseases such as diabetes mellitus, aging, cardiovascular disease, cancer and other degenerative diseases (Pham-Huy et al., 2008[28]; Phaniendra et al., 2015[29]). Therefore, compounds or substances acting as antioxidants have drawn considerable attention in finding new medicinal agents (Carocho and Ferreira, 2013[5]; Wojcik et al., 2010[44]). The target compounds could be achieved based on the known pharmacophores and functional groups of drugs/bioactive compounds (Hughes et al., 2011[15]; Lounnas et al., 2013[21]). A variety of bioactive compounds has been reported i.e., sulfonamide and its derivatives (Khan et al., 2018[17]).

Sulfonamide (SO_2_NH) is an important functional pharmacophore found in drugs and bioactive compounds. For example, sulfa drugs were used for the prevention and cure of bacterial infections (Smith and Powell, 2000[37]). Chemically modified core structures of sulfonamide have been reported (Bhat et al., 2005[4]; Carta et al., 2012[6]) to improve pharmacological properties such as antimicrobial (Durgun et al., 2017[12]), anticancer (Scozzafava et al., 2003[35]), antiviral (Supuran et al., 2004[38]), and antimalarial (Ugwu et al., 2017[42]) activities as well as cyclooxygenase-2 inhibitors (Gedawy et al., 2020[14]). In addition, 4-substituted (R = NO_2_, OCH_3_, CH_3_, Cl) benzenesulfonamides and related compounds were synthesized and investigated for antioxidant, antimicrobial, anticancer and antifertility activities (Doungsoongnuen et al., 2011[10]; Pholpramool et al., 1991[30]; Temcharoen et al., 1994[40]). Furthermore, sulfonamides containing coumarin moieties were reported to exhibit antioxidant activity (Saeedi et al., 2014[34]).

Based on the literature, 4-acetamidobenzenesulfonamide analogs are attractive target molecules to be explored as antimicrobials, antioxidants and computational study approaching their physicochemical properties related to biological activities. 

Quantitative structure-activity relationship (QSAR) has been a common computational technique for model construction using for elucidation of the structure-activity relationship (SAR) of compounds (Nantasenamat et al., 2010[22]; Prachayasittikul et al., 2015[33]).

This study aims to synthesize acetamidosulfonamide derivatives and investigate for antioxidant and antimicrobial activities as well as QSAR study using multiple linear regression (MLR). 

## Materials and Methods

### Chemistry 

Column chromatography was carried out using silica gel 60 (70-230 mesh ASTM). Analytical thin-layer chromatography (TLC) was performed on silica gel 60 F_254_ aluminum sheets. ^1^H- and ^13^C-NMR spectra were recorded on a Bruker AVANCE 300 NMR spectrometer. FTIR spectra were obtained using a universal attenuated total reflectance attached on a Perkin-Elmer Spectrum One spectrometer. High resolution mass spectra (HRMS) were recorded on a Bruker Daltonics (microTOF). Melting points were determined using a Griffin melting point apparatus and were uncorrected. Chemicals and reagents were purchased: vitamin E, DPPH (2,2-diphenyl-1-picrylhydrazyl), nitro blue tetrazolium (NBT) salt, L-methionine, riboflavin, Triton-100 and superoxide dismutase (SOD, bovine erythrocytes) from Sigma, USA. DMSO (dimethyl sulfoxide, 99.9 %) from RCI Labscan, Thailand; methanol from Merck, Germany; ampicillin, ciprofloxacin and tetracycline from Sigma, USA; Muller Hinton Broth and Muller Hinton Agar from Becton Dickinson, USA; and sodium chloride from Merck, German. Solvents were analytical grades.

### General procedure for the synthesis of sulfonamides (1-16)

A solution of 4-acetamidobenzenesulfonyl chloride (5 mmol) in dichloromethane (30 mL) was added in a dropwise manner to a stirred mixture of amine (5 mmol) and sodium carbonate (7 mmol) in dichloromethane (20 mL). The reaction mixture was stirred at room temperature until completion of reaction (monitored by TLC), and added distilled water (20 mL). The organic phase was separated and the aqueous phase was extracted with dichloromethane (2 × 30 mL). The organic extracts were combined and washed with water (30 mL). The organic layer was dried over anhydrous sodium sulfate (anh. Na_2_SO_4_), filtered and evaporated to dryness under reduced pressure. The crude product was further purified by column chromatography on silica gel. In case of sulfonamide **7**, it was synthesized using 4-acetamidobenzenesulfonyl chloride (10 mmol), piperazine (5 mmol) and sodium carbonate (14 mmol).

### N-(4-(N-benzylsulfamoyl)phenyl)acetamide (1) (De Luca and Giacomelli, 2008)

White solid. 85 % yield; mp 150-152^ o^C; IR (UATR) cm^-1^: 3329, 3271, 1681, 1592, 1531, 1320, 1154. ^1^H NMR (300 MHz, DMSO-d_6_) δ 2.08 (s, 3H, C*H*_3_CO), 3.93 (d, J = 6.2 Hz, 2H, C*H*_2_N), 7.14-7.31 (m, 5H, Ar*H*), 7.71 (d, J = 9.4 Hz, 2H, Ar*H*), 7.73 (d, J = 9.4 Hz, 2H, Ar*H*), 7.99 (t, J = 6.2 Hz, 1H, N*H*SO_2_), 10.30 (s, 1H, N*H*CO). ^13^C NMR (75 MHz, DMSO-d_6_) δ 23.9, 46.0, 118.5, 126.9, 127.4, 128.0, 134.4, 137.6, 142.5, 168.8. TOF-MS *m*/*z*: 305.0968 (Calcd for C_15_H_17_N_2_O_3_S: 305.0954). 

### N-(4-(N-(pyridin-2-ylmethyl)sulfamoyl)phenyl)acetamide (2) 

Pale brown solid. 70 % yield; mp 116-118 °C; IR (UATR) cm^-1^: 3330, 3232, 1673, 1592, 1534, 1319, 1151. ^1^H NMR (300 MHz, DMSO-d_6_) δ 1.99 (s, 3H, C*H*_3_CO), 3.96 (d, J = 6.3 Hz, 2H, C*H*_2_N), 7.14 (dd, J = 7.4, 5.0 Hz, 1H, Py*H*), 7.26 (d, J = 7.8 Hz, 1H, Py*H*), 7.58-7.70 (m, 5H, Ar*H*_4_, Py*H*), 8.03 (t, J = 6.3 Hz, 1H, N*H*SO_2_), 8.34 (d, J = 4.7 Hz, 1H, Py*H*), 10.21 (s, 1H, N*H*CO). ^13^C NMR (75 MHz, DMSO-d_6_) δ 24.6, 48.4, 119.0, 122.1, 122.8, 128.1, 134.6, 137.1, 143.2, 149.2, 157.7, 169.4. TOF-MS *m*/*z*: 328.0731 (Calcd for C_14_H_15_N_3_NaO_3_S: 328.0726).

### N-(4-(N-(pyridin-3-ylmethyl)sulfamoyl)phenyl)acetamide (3) 

Pale yellow solid. 74 % yield; mp 179-180^ o^C; IR (UATR) cm^-1^: 3178, 1681, 1591, 1543, 1322, 1159. ^1^H NMR (300 MHz, DMSO-d_6_) δ 2.08 (s, 3H, C*H*_3_CO), 3.99 (s, 2H, C*H*_2_N), 7.28 (dd, J = 7.7, 4.8 Hz, 1H, Py*H*), 7.62 (d, J = 7.8 Hz, 1H, Py*H*), 7.70 (d, J = 9.3 Hz, 2H, Ar*H*), 7.73 (d, J = 9.4 Hz, 2H, Ar*H*), 8.36-8.45 (m, 2H, Py*H*), 10.33 (br s, 1H, N*H*CO). ^13^C NMR (75 MHz, DMSO-d_6_) δ 24.1, 43.7, 118.6, 123.3, 127.7, 133.4, 134.2, 135.4, 142.8, 148.3, 148.9, 169.0. TOF-MS *m*/*z*: 328.0724 (Calcd for C_14_H_15_N_3_NaO_3_S: 328.0726). 

### N-(4-(N-((tetrahydrofuran-2-yl)methyl)sulfamoyl)phenyl)acetamide (4)

White solid. 79 % yield; mp 130-132^ o^C; IR (UATR) cm^-1^: 3329, 1691, 1592, 1533, 1315, 1152. ^1^H NMR (300 MHz, DMSO-d_6_) δ 1.40-1.88 (m, 4H, 2 × C*H*_2_), 2.06 (s, 3H, C*H*_3_CO), 2.72 (t, J = 6.2 Hz, 2H, C*H*_2_N), 3.47-3.81 (m, 3H, C*H*_2_O, C*H*O), 7.56 (t, J = 6.2 Hz, 1H, N*H*SO_2_), 7.69 (d, J = 9.0 Hz, 2H, Ar*H*), 7.73 (d, J = 9.2 Hz, 2H, Ar*H*), 10.31 (s, 1H, N*H*CO). ^13^C NMR (75 MHz, DMSO-d_6_) δ 24.1, 25.1, 28.5, 46.6, 67.3, 77.0, 118.7, 127.7, 134.5, 142.7, 169.1. TOF-MS *m*/*z*: 299.1055 (Calcd for C_13_H_19_N_2_O_4_S: 299.1060). 

### N-(4-((4-methylpiperazin-1-yl)sulfonyl)- phenyl)acetamide (5) (Barbosa et al., 2009)

White solid. 78 % yield; mp 286-287^ o^C; IR (UATR) cm^-1^: 3327, 1682, 1590, 1530, 1316, 1151. ^1^H NMR (300 MHz, DMSO-d_6_) δ 2.07 (s, 3H, C*H*_3_CO), 2.10 (s, 3H, C*H*_3_N), 2.32 (t, J = 4.7 Hz, 4H, 2 × C*H*_2_), 2.83 (br t, 4H, 2 × C*H*_2_), 7.63 (d, J = 8.9 Hz, 2H, Ar*H*), 7.80 (d, J = 8.8 Hz, 2H, Ar*H*), 10.43 (s, 1H, N*H*CO). ^13^C NMR (75 MHz, DMSO-d_6_) δ 24.2, 45.4, 45.8, 53.6, 118.8, 128.8, 128.9, 143.6, 169.4. TOF-MS *m*/*z*: 298.1225 (Calcd for C_13_H_20_N_3_O_3_S: 298.1220). 

### N-(4-((4-benzylpiperazin-1-yl)sulfonyl)phenyl)acetamide (6)

White solid. 81 % yield; mp 189-190^ o^C; IR (UATR) cm^-1^: 3337, 1680, 1590, 1528, 1314, 1160. ^1^H NMR (300 MHz, DMSO-d_6_) δ 2.18 (s, 3H, C*H*_3_CO), 2.49 (br t, 4H, 2 × C*H*_2_), 2.93 (br t, 4H, 2 × C*H*_2_), 3.53 (s, 2H, C*H*_2_Ph), 7.25-7.40 (m, 5H, Ar*H*), 7.73 (d, J = 8.8 Hz, 2H, Ar*H*), 7.90 (d, J = 8.8 Hz, 2H, Ar*H*), 10.48 (s, 1H, N*H*CO). ^13^C NMR (75 MHz, DMSO-d_6_) δ 24.0, 45.8, 51.3, 61.3, 118.6, 126.9, 128.1, 128.6, 128.7, 137.6, 143.4, 169.0. TOF-MS *m*/*z*: 374.1531 (Calcd for C_19_H_24_N_3_O_3_S: 374.1533). 

### N,N'-((piperazine-1,4-disulfonyl)bis(4,1-phenylene))diacetamide (7)

White solid. 66 % yield; mp 309-310^ o^C; IR (UATR) cm^-1^: 3265, 1684, 1588, 1538, 1345, 1160. ^1^H NMR (300 MHz, DMSO-d_6_) δ 2.09 (s, 6H, 2 × C*H*_3_CO), 2.92 (s, 8H, 4 × C*H*_2_), 7.61 (d, J = 8.7 Hz, 4H, Ar*H*), 7.79 (d, J = 8.7 Hz, 4H, Ar*H*), 10.39 (s, 2H, 2 × N*H*CO). ^13^C NMR (75 MHz, DMSO-d_6_) δ 24.2, 45.2, 118.8, 128.3, 128.8, 143.7, 169.3. TOF-MS *m*/*z*: 503.1032 (Calcd for C_20_H_24_N_4_NaO_6_S_2_: 503.1030). 

### N-(4-(morpholinosulfonyl)phenyl)- acetamide (8) (Barbosa et al., 2009)

White solid. 73 % yield; mp 159-160^ o^C; IR (UATR) cm^-1^: 3303, 1677, 1590, 1526, 1343, 1161. ^1^H NMR (300 MHz, DMSO-d_6_) δ 2.08 (s, 3H, C*H*_3_CO), 2.81 (t, J = 4.6 Hz, 4H, 2 × C*H*_2_), 3.60 (t, J = 4.6 Hz, 4H, 2 × C*H*_2_), 7.66 (d, J = 8.8 Hz, 2H, Ar*H*), 7.83 (d, J = 8.8 Hz, 2H, Ar*H*), 10.39 (s, 1H, N*H*CO). ^13^C NMR (75 MHz, DMSO-d_6_) δ 24.2, 45.9, 65.3, 118.7, 127.8, 128.9, 143.6, 169.2. TOF-MS *m*/*z*: 307.0734 (Calcd for C_12_H_16_N_2_NaO_4_S: 307.0723). 

### N-(4-((3,4-dihydroisoquinolin-2(1H)-yl)sulfonyl)phenyl)acetamide (9) (Pagliero et al., 2011)

White solid. 73 % yield; mp 181-182^ o^C; IR (UATR) cm^-1^: 3301, 1678, 1590, 1527, 1337, 1160. ^1^H NMR (300 MHz, DMSO-d_6_) δ 2.07 (s, 3H, C*H*_3_CO), 2.83 (t, J = 5.6 Hz, 2H, C4-IQ*H*), 3.24 (t, J = 5.8 Hz, 2H, C3- IQ*H*), 4.14 (s, 2H, C1- IQ*H*), 7.05-7.15 (m, 4H, IQ*H*), 7.73 (d, J = 8.9 Hz, 2H, Ar*H*), 7.79 (d, J = 9.0 Hz, 2H, Ar*H*), 10.35 (s, 1H, N*H*CO). ^13^C NMR (75 MHz, DMSO-d_6_) δ 24.6, 28.5, 44.0, 47.7, 119.2, 126.6, 126.9, 127.1, 129.1, 129.8, 132.1, 133.5, 143.9, 169.6. TOF-MS *m*/*z*: 353.0942 (Calcd for C_17_H_18_N_2_NaO_3_S: 353.0942). 

### N-(4-(N-cyclohexylsulfamoyl)phenyl)- acetamide (10)

White solid. 70 % yield; mp 213-215^ o^C; IR (UATR) cm^-1^: 3328, 3273, 1694, 1596, 1539, 1312, 1147. ^1^H NMR (300 MHz, DMSO-d_6_) δ 0.90-1.60 (m, 10H, 5 × C*H*_2_), 2.07 (s, 3H, C*H*_3_CO), 2.86 (br s, 1H, C*H*N), 7.47 (d, J = 7.3 Hz, 1H, N*H*SO_2_), 7.72 (s, 4H, Ar*H*), 10.30 (s, 1H, N*H*CO). ^13^C NMR (75 MHz, DMSO-d_6_) δ 24.1, 24.4, 24.9, 33.3, 52.1, 118.7, 127.4, 136.0, 142.5, 169.1. TOF-MS *m*/*z*: 319.1096 (Calcd for C_14_H_20_N_2_NaO_3_S: 319.1087). 

### N-(4-(N-cyclopentylsulfamoyl)phenyl)- acetamide (11)

White solid. 69 % yield; mp 179-180^ o^C; IR (UATR) cm^-1^: 3274, 1672, 1594, 1539, 1321, 1161. ^1^H NMR (300 MHz, DMSO-d_6_) δ 1.15-1.64 (m, 8H, 4 × C*H*_2_), 2.07 (s, 3H, C*H*_3_CO), 3.25-3.35 (m, 1H, C*H*N), 7.47 (d, J = 7.1 Hz, 1H, N*H*SO_2_), 7.70 (d, J = 9.2 Hz, 2H, Ar*H*), 7.74 (d, J = 9.2 Hz, 2H, Ar*H*), 10.29 (s, 1H, N*H*CO). ^13^C NMR (75 MHz, DMSO-d_6_) δ 23.2, 24.5, 33.1, 54.7, 119.1, 128.1, 135.7, 142.9, 169.7. TOF-MS *m*/*z*: 305.0937 (Calcd for C_13_H_18_N_2_NaO_3_S: 305.0930). 

### N-(4-(N-phenylsulfamoyl)phenyl)acetamide (12) (Silveira et al., 2017)

White solid. 70 % yield; mp 200-202^ o^C; IR (UATR) cm^-1^: 3239, 1672, 1591, 1542, 1333, 1166. ^1^H NMR (300 MHz, DMSO-d_6_) δ 2.04 (s, 3H, C*H*_3_CO), 6.99 (t, J = 7.1 Hz, 1H, Ar*H*), 7.04 (d, J = 8.3 Hz, 2H, Ar*H*), 7.19 (t, J = 7.7 Hz, 2H, Ar*H*), 10.28 (s, 1H, N*H*CO). ^13^C NMR (75 MHz, DMSO-d_6_) δ 24.4, 119.1, 120.6, 124.6, 128.4, 129.6, 133.5, 138.1, 143.4, 169.8. TOF-MS *m*/*z*: 291.0807 (Calcd for C_14_H_15_N_2_O_3_S: 291.0798). 

### N-(4-(N-phenethylsulfamoyl)phenyl)- acetamide (13)

White solid. 85 % yield; mp 142-144^ o^C; IR (UATR) cm^-1^: 3325, 3273, 1677, 1592, 1530, 1318, 1152. ^1^H NMR (300 MHz, DMSO-d_6_) δ 2.04 (s, 3H, C*H*_3_CO), 2.60 (t, J = 7.4 Hz, 2H, C*H*_2_Ph), 2.88 (q, J = 6.8 Hz, 2H, C*H*_2_N), 7.02-7.25 (m, 5H, Ar*H*), 7.49 (d, J = 7.3 Hz, 1H, N*H*SO_2_), 7.65 (s, 4H, Ar*H*), 10.36 (s, 1H, N*H*CO). ^13^C NMR (75 MHz, DMSO-d_6_) δ 24.2, 35.4, 44.4, 119.6, 126.8, 128.1, 128.8, 129.0, 134.4, 138.9, 142.7, 170.7. TOF-MS *m*/*z*: 341.0927 (Calcd for C_16_H_18_N_2_NaO_3_S: 341.0930). 

### N-(4-(N-(3,4-dimethoxyphenethyl)- sulfamoyl)phenyl)acetamide (14)

White solid. 86 % yield; mp 134-136^ o^C; IR (UATR) cm^-1^: 3327, 1682, 1592, 1516, 1322, 1149. ^1^H NMR (300 MHz, DMSO-d_6_) δ 2.07 (s, 3H, C*H*_3_CO), 2.56 (t, J = 7.4 Hz, 2H, C*H*_2_Ph), 2.90 (q, J = 6.9 Hz, 2H, C*H*_2_N), 3.68, 3.69 (2s, 6H, 2 × OC*H*_3_), 6.62 (dd, J = 8.1, 1.6 Hz, 1H, Ar*H*), 6.70 (d, J = 1.6 Hz, 1H, Ar*H*), 6.79 (d, J = 8.2 Hz, 1H, Ar*H*), 7.52 (t, J = 5.7 Hz, 1H, N*H*SO_2_), 7.68 (d, J = 9.0 Hz, 2H, Ar*H*), 7.73 (d, J = 9.0 Hz, 2H, Ar*H*), 10.31 (s, 1H, N*H*CO). ^13^C NMR (75 MHz, DMSO-d_6_) δ 24.5, 35.2, 44.7, 55.9, 56.0, 112.4, 113.0, 119.1, 121.0, 128.1, 131.6, 134.6, 143.1, 147.8, 149.1, 169.5. TOF-MS *m*/*z*: 401.1145 (Calcd for C_18_H_22_N_2_NaO_5_S: 401.1142). 

### N-(4-(N-(2-(pyridin-2-yl)ethyl)sulfamoyl)phenyl)acetamide (15)

White solid. 78 % yield; mp 138-140^ o^C; IR (UATR) cm^-1^: 3238, 1686, 1592, 1541, 1319, 1164. ^1^H NMR (300 MHz, DMSO-d_6_) δ 2.07 (s, 3H, C*H*_3_CO), 2.81 (t, J = 7.4 Hz, 2H, C*H*_2_Py), 3.06 (q, J = 6.8 Hz, 2H, C*H*_2_N), 7.15-7.22 (m, 2H, Py*H*), 7.58 (t, J = 5.8 Hz, 1H, N*H*SO_2_), 7.62-7.77 (m, 5H, Ar*H*_4_, Py*H*), 8.42 (d, J = 4.2 Hz, 1H, Py*H*), 10.31 (s, 1H, N*H*CO). ^13^C NMR (75 MHz, DMSO-d_6_) δ 24.5, 37.7, 42.7, 119.2, 122.1, 123.7, 128.1, 134.5, 136.9, 143.1, 149.5, 158.8, 169.5. TOF-MS *m*/*z*: 320.1055 (Calcd for C_15_H_18_N_3_O_3_S: 320.1063). 

### N-(4-(N-(2-(diethylamino)ethyl)- sulfamoyl)phenyl)acetamide (16)

White solid. 71 % yield; mp 76-78^ o^C; IR (UATR) cm^-1^: 3322, 3273, 1681, 1592, 1532, 1317, 1153. ^1^H NMR (300 MHz, DMSO-d_6_) δ 0.84 (t, J = 7.2 Hz, 6H, 2 × C*H*_3_), 2.06 (s, 3H, C*H*_3_CO), 2.29-2.40 (m, 6H, 3 × C*H*_2_), 2.74 (t, J = 7.3 Hz, 2H, C*H*_2_), 7.69 (d, J = 9.0 Hz, 2H, Ar*H*), 7.74 (d, J = 9.1 Hz, 2H, Ar*H*), 10.33 (s, 1H, N*H*CO). ^13^C NMR (75 MHz, DMSO-d_6_) δ 12.2, 24.4, 41.4, 47.1, 52.3, 119.3, 128.0, 135.0, 143.1, 169.5. TOF-MS *m*/*z*: 314.1528 (Calcd for C_14_H_24_N_3_O_3_S: 314.1533).

### Biological activity evaluation

#### Antioxidant activity assays

Radical scavenging activity (RSA) was performed by adding 1 mL of 2,2-diphenyl-1-picrylhydrazyl (DPPH) in methanol (0.1 mM) to the tested compounds (dissolved in DMSO) with the final concentration of 300 μg/mL, and the mixture was kept in the dark for 30 min. The absorbance at 517 nm was measured using UV-Visible spectrophotometer (UV-1610, Shimadzu). The DPPH (stable purple compound) reacted with antioxidants to give light-yellow color product of 1,1-diphenyl-2-picrylhydrazine (Worachartcheewan et al., 2012[47]). Vitamin E was used as a control and DMSO was used as a blank reaction. The RSA ( %) was computed using equation (1):


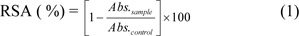


where *Abs*._control_ is the absorbance of the control reaction, and *Abs.*_sample_ is the absorbance of the tested compound. 

Superoxide scavenging activity was evaluated using superoxide dismutase (SOD) assay by measuring nitro blue tetrazolium (NBT) reduction (Piacham et al., 2003[31]). The stock solution containing 27 mL of HEPES buffer (50 mM, pH 7.8), 1.5 mL of L-methionine (30 mg/mL), 1 mL of NBT (1.41 mg/mL) and 750 μL of Triton X-100 (1 % wt) was prepared. The 1 mL of stock solution was added to the solution of tested compounds (**1**-**16**, dissolved in DMSO) with the final concentration of 300 μg/mL. The reaction was initially started by adding 10 μL of riboflavin (44 mg/mL), and followed by illumination under a Philips Classic Tone lamp (60 W) in a light box for 7 min. The absorbance at 550 nm of the reaction was measured using UV-Visible spectrophotometer (UV-1610, Shimadzu). SOD from bovine erythrocytes was used as a control, and DMSO was used as a blank reaction. The SOD activity (%) was calculated using equation (2):


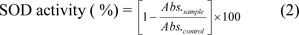


where *Abs*._control_ is the absorbance of the control reaction, and *Abs.*_sample_ is the absorbance of the tested compound. 

If compounds exhibited antioxidant (RSA and SOD) activities greater than 50 % at 300 μg/mL, their IC_50_ values were performed by linear regression plot between %RSA or %SOD activity and compound concentrations. 

#### Antimicrobial activity assay

The tested compounds were investigated for antimicrobial activity using the agar-dilution method (Baron et al., 1994[3]). The compounds were prepared and then transferred to Muller Hinton (MH) agar to obtain the final concentration of 4-256 μg/mL. The microorganisms were cultured in MH broth at 37 °C overnight, and then suspended in 0.9 % normal saline solution. Turbidity of microorganism was adjusted to give a cell density of 1×10^8^ CFU/mL compared to 0.5 McFarland turbidity standard. The microorganisms were inoculated onto the agar plates containing a variety of compound concentrations using a multipoint inoculator, and incubated at 37 °C for 24-48 h. The control plate containing DMSO, MH broth and antibacterial agents (i.e., ampicillin, ciprofloxacin and tetracycline) were also parallelly performed. The inhibition of microbial cell growth was determined as the minimum inhibitory concentration (MIC), which is the lowest concentration to completely inhibit the growth of microorganisms. The twenty-six strains of microorganisms for the assay consisted of reference strains and clinical isolates; gram positive bacteria: *Staphylococcus aureus* ATCC 29213, *Staphylococcus aureus* ATCC 25923, *Staphylococcus epidermidis* ATCC 12228,* Enterococcus faecalis* ATCC 29212, *Enterococcus faecalis *ATCC 33186, *Micrococcus luteus* ATCC 10240, *Corynebacterium diphtheriae* NCTC 10356, *Bacillus subtilis* ATCC 6633, *Listeria monocytogenes*, *Bacillus cereus*; gram negative bacteria: *Escherichia coli* ATCC 25922*, Klebsiella pneumoniae* ATCC 700603, *Serratia marcescens* ATCC 8100, *Salmonella typhimurium* ATCC 13311, *Shewanella putrefaciens* ATCC 8071, *Achromobacter xylosoxidans* ATCC 2706, *Pseudomonas aeruginosa* ATCC 27853, *Pseudomonas stutzeri* ATCC 17587, *Shigella dysenteriae*, *Salmonella enteritidis*, *Morganella morganii*, *Aeromonas hydrophila*,* Citrobacter freundii*, *Plesiomonas shigelloides* and diploid fungus (yeast): *Candida albicans* ATCC 90028, *Saccharomyces cerevisiae* ATCC 2601.

### QSAR analysis

#### Data set

The bioactive sulfonamide derivatives (**1**-**16**) were used as a data set to obtain significant descriptors (independent variables) correlating with biological properties (dependent variable) for development of QSAR models.

### Calculation of quantum chemical and molecular descriptors

Chemical structures of the sulfonamides were constructed by Chemdraw Pro13 software (PerkinElmer, USA) which subjected to GaussView (Dennington et al., 2003[9]) software and further geometrically optimized by Gaussian 09, Revision A.02 (Frisch et al., 2009[13]) at the semi-empirical level using Austin Model 1 (AM1), then followed by density functional theory (DFT) calculation using Becke's three-parameter hybrid method, and the Lee-Yang-Parr correlation functional (B3LYP) together with the 6-31 g(d) basis set. The low-energy conformers of the compounds were obtained and output files were consequently employed to calculate quantum chemical descriptors including the total energy (*E_total_*) of the molecule, the highest occupied molecular orbital energy (*E_HOMO_*), the lowest unoccupied molecular orbital energy (*E_LUMO_*), the total dipole moment (*µ*) of the molecule, the electron affinity (EA), the ionization potential (IP), the energy difference of HOMO and LUMO (HOMO-LUMO_Gap_), Mulliken electronegativity (χ), hardness (η), softness (*S*), electrophilicity (ω), electrophilic index (ω*_i_*) and the mean absolute atomic charge (*Q_m_*) (Karelson et al., 1996[16]; Parr et al., 1978[25], 1999[27]; Parr and Pearson, 1983[26]; Thanikaivelan et al., 2000[41]). The output files were used as the input data for calculating a set of 3,224 molecular descriptors using Dragon software, version 5.5 (Talete, 2007[39]) to give the following 22 categories: 48 constitutional descriptors, 119 topological descriptors, 47 feature selection of descriptors walk and path counts, 33 connectivity indices, 47 information indices, 96 2D autocorrelation, 107 edge adjacency indices, 64 burden eigenvalues, 21 topological charge indices, 44 eigenvalue-based indices, 41 randic molecular profiles, 74 geometrical descriptors, 150 RDF descriptors, 160 3D-MoRSE descriptors, 99 WHIM descriptors, 197 GETAWAY descriptors, 154 functional group counts, 120 atom-centred fragments, 14 charge descriptors, 29 molecular properties, 780 2D binary fingerprints and 780 2D frequency fingerprints.

### Feature selection of descriptors

A volume of 3,224 molecular descriptors was filtered to reduce multi-collinear and redundant descriptor variables. Constant values and pairs of variables with correlation coefficient greater than 0.99 were removed in Dragon software. The remaining 1,387 molecular descriptors were combined with a set of 13 quantum chemical descriptors. The descriptors correlated with their activities were selected using automatic variable selection procedure (CfsSubsetEval combined with the BestFirst) in Waikato Environment for Knowledge Analysis (Weka) software, version 3.4.5 (Witten et al., 2011[43]), and following by stepwise multiple linear regression (MLR) (SPSS statistics 18.0, SPSS Inc., USA). The obtained descriptors were determined for the intercorrelation matrix among each descriptor by Pearson's correlation coefficient (*r*) using SPSS statistics 18.0 (SPSS Inc., USA). The cutoff of |*r*| ≥ 0.8 was assigned to describe collinearly between the descriptors. 

### Multiple linear regression for QSAR models construction

QSAR models were carried out by multiple linear regression (MLR) using Weka software, version 3.4.5 (Witten et al., 2011[43]). Significant descriptors were used as independent variables (X) and their biological activities were used as dependent variable (Y). The QSAR models were constructed by equation (3): 


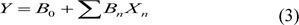


where *Y* is the biological activities of the compounds, *B*_0 _is the intercept, and *B_n_* are the regression coefficients of the descriptors *X_n_*.

### Generation of dataset 

The data set was divided into two sets composed of training and testing sets. The training set was used to construct the QSAR models, whereas the testing set was employed to evaluate the model. The testing set was performed by means of leave-one-out cross validation (LOO-CV) which one sample was left out from the data set (*N*) to be used as the testing set while the remaining set (*N-1*) was used as the training set. This was iteratively continued until every sample had a chance to be the testing set. The biological activities of the compounds were predicted using equation (3).

### Evaluation of QSAR models

Predictive performance of the constructed QSAR models was evaluated by statistical analysis. Squared correlation coefficient (*R*^2^*_Tr_*) for training, and cross-validated *Q^2^* (*Q*^2^*_LOO-CV_*) for LOO-CV sets were used to measure a relative correlation of the predicted and the experimental values. Furthermore, root mean square error (RMSE) was used to determine the predictive error of the model for training (*RMSE_Tr_*) and LOO-CV (*RMSE_LOO-CV_*) sets (Nantasenamat et al., 2010[22]).

## Results and Discussion

### Chemistry

#### N-sulfonylation

**Figure 1:**
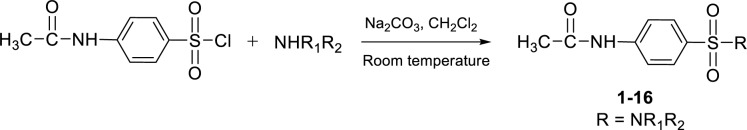
Synthesis of sulfonamide derivatives (1-16)

Sixteen sulfonamides (**1**-**16**) were synthesized in 66-86 % yields by sulfonylation of various amines with 4-acetamidobenzenesulfonyl chloride containing base, sodium carbonate (Na_2_CO_3_), in dichloromethane (CH_2_Cl_2_) at room temperature (Figure 1(Fig. 1)). 

Structures of the sulfonamides (**1**-**16**) were characterized by their HRMS, ^1^H and ^13^C NMR and IR spectra. All sulfonamides had molecular ion peaks corresponding to their molecular formula. ^1^H NMR spectra of *N*-acetyl compounds (**1**-**16**) displayed the characteristic signals of CH_3_CO and NHCO protons as two singlets at *δ *in the range of 1.9-2.2 and 10.2-10.5 ppm, respectively whereas their ^13^C NMR spectra showed carbonyl groups at *δ *168-171 ppm. Infrared spectra of the acetamides (**1**-**16**) gave N-H and C=O absorptions at around 3170-3340 and 1670-1700 cm^−1^, respectively. 

These sulfonamides have a common 4-acetamidobenzenesulfonyl core structure bearing various amino substituents (R) as shown in Table 1(Tab. 1). The R group can be methylamino (**1**-**4**, group A), ethylamino (**13**-**16**, group B), cyclic amino (**5**-**9**, group C) and ring substituted amino (**10**-**12**, group D) moieties. 

### Biological activities

#### Radical scavenging activity 

The results revealed that most sulfonamides displayed radical scavenging activity (RSA) toward the DPPH radical in 0.41-4.62 % range at concentration of 300 µg/mL, except for compounds **3**, **5** and **16** had no activity (Table 1(Tab. 1)). The activity of compounds is dependent on the functional (R) groups of the core structure (Table 1(Tab. 1)). The most potent compound **15** (R = NHCH_2_CH_2_-2-pyridyl) exhibited 4.62 % RSA, whereas R = NHCH_2_C_6_H_5_ (**1**) showed the lowest activity (0.42 % RSA).

### Superoxide scavenging activity 

Superoxide scavenging (SOD) assay was performed to show the ability of compounds to inhibit the photoreduction of NBT. Most sulfonamides had the SOD activity (25.22-38.54 %) at 300 µg/mL, except for compounds **5** and **7** with no activity (Table 1(Tab. 1)). Interestingly, compound **15** displayed the highest antioxidant activities in SOD (38.54 %) and DPPH (4.62 %) assays.

### Antimicrobial activity

The sulfonamides (**1**-**16**) were investigated for antimicrobial activity using the agar dilution against gram positive bacteria, gram negative bacteria and diploid fungi from reference strains and clinical isolates. It was found that all compounds were inactive at concentration of 256 µg/mL.

### QSAR analysis 

QSAR is a potential model for exploring the relationship between physicochemical properties and bioactivities of compounds as well as rational design of new bioactive compounds. This has been demonstrated in compounds with antimicrobial (Cherdtrakulkiat et al., 2020[7]), antioxidant (Worachartcheewan et al., 2014[46]), anticancer (Leechaisit et al., 2019[19]) and antiviral (Worachartcheewan et al., 2019[48]) activities. To construct the model, the quantum chemical and molecular descriptors of the compounds were computed using Gaussian 09 and Dragon softwares, respectively (Alyar and Karacan, 2009[1]; Nantasenamat et al., 2013[23]; Pingaew et al., 2015[32]; Worachartcheewan et al., 2011[45]). The obtained 3,224 descriptors from the Dragon software were initially filtered to remove multi-collinear and redundant descriptor variables. This process gave 1,387 descriptors which were further combined with 13 quantum chemical descriptors from the Gaussian 09 software to provide the final set of 1,400 descriptors. The important descriptors correlated with their antioxidant activities were achieved using automatic variable selection procedure (CfsSubsetEval combined with the BestFirst) in WEKA software and following by stepwise MLR. Six descriptors for the QSAR model of RSA included G2u, Lop, RDF120m, N-067, H1v and RDF045m, whereas 6 descriptors for the QSAR model of SOD activity were RDF120m, Mor27u, R7u+, BELp5, G3m and BELm8. The definition and type of descriptors are listed in Table 2(Tab. 2). The intercorrelation matrix was performed by Pearson's correlation coefficient (*r*) in which each descriptor was explored using independent descriptors with value of |*r*| < 0.8 in both RSA and SOD activities (Tables S1 and S2, Supplementary information). 

The constructed QSAR model for RSA was generated from Eq. (3) using 6 significant descriptors as independent variables, and %RSA as dependent variable. The 13 active sulfonamides (**1**,** 2**,** 4**,** 6-15)** were used as data set for training and LOO-CV sets to build up and evaluate the QSAR model (Eq. (4).


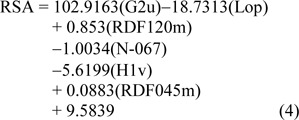


**Figure 2:**
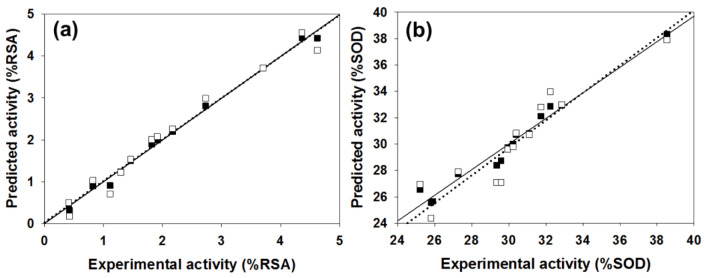
Plot of the experimental versus predicted RSA (a) and SOD (b) activities using MLR method. The training set was represented by black squares and solid line, and the leave-one-out cross-validation set was assigned by white squares and dotted line

Table 3(Tab. 3) showed correlation coefficients (*R^2^* and *Q^2^*) and root mean square error (RMSE) of training (*Tr*) set as 0.9948 and 0.0985, respectively, and of leave-one-out cross validation (LOO-CV) set as 0.9708 and 0.5705, respectively. The correlation coefficient in training and LOO-CV sets exhibited high correlation. It indicated reliability of statistical quality with correlation coefficient of training set (*R^2^*) > 0.6 and LOO-CV set (*Q^2^*) >0.5 (Nantasenamat et al., 2010[22]), but low RMSE values. This suggested that the six descriptors (Table 2(Tab. 2)) were related with antioxidant activity, and their numerical values are presented in Table 4(Tab. 4). The plot between experimental and predicted (%RSA) activity is shown in Figure 2a(Fig. 2). Considering regression coefficients in Eq. (4), descriptors G2u, RDF120m and RDF045m showed positive values of 102.9163, 0.853 and 0.0883, respectively, while Lop, N-067 and H1v descriptors had negative values of -18.7313, -1.0034 and -5.6199, respectively. This implied that the positive values of regression coefficients provided the positive effect which is responsible for increasing the activity, on the other hand, the negative values of regression coefficients reduce the activity. 

Similarly, the QSAR model of SOD activity was constructed using 14 active sulfonamides (**1**-**4**, **6**, **8**-**16**) (Table 1(Tab. 1)), and 6 significant descriptors. The generated QSAR model was obtained as shown in equation (5):


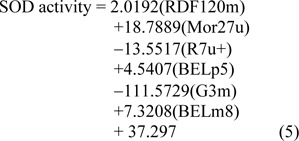


The statistical results of training set displayed *R*^2^*_Tr_* and *RMSE_Tr_* values of 0.9714 and 0.2306, whereas *Q*^2^*_LOO-CV _*and *RMSE_LOO-CV_* of LOO-CV set were 0.8753 and 1.3571, respectively (Table 3(Tab. 3)). The statistical quality provided high correlation between the experimental and the predicted values, but low RMSE values in the training and LOO-CV sets. The six descriptors in Eq. (5) were correlated with the SOD activity, and the numerical values are shown in Table 5(Tab. 5). The plot of experimental and predicted (%SOD) activity is shown in Figure 2b(Fig. 2). The regression coefficients including RDF120m, More27u, BELp5 and BELm8 descriptors have positive values of 2.0192, 18.7889, 4.5407 and 7.3208, respectively, whereas R7u+ and G3m descriptors showed negative values of -13.5517 and -111.5729, respectively. The positive values of regression coefficients impact the increase activity, but the negative values diminished the activity.

### Structure-activity relationship and rational design

Considering the effect of substituted (R) group on antioxidant activity, it was found that R = NHCH_2_CH_2_-2-pyridyl (**15**) exerted the highest RSA (4.62 %) and SOD (38.54 %) activities. When R = *N*-methyl piperazine, compound **5** displayed no RSA and SOD activities.

Group A compounds (**1**-**4**) showed SOD (25.22-30.40 %) and RSA (0.42-1.82 %) activities, compound **2** (R = NHCH_2_-2-pyridiyl) displayed the highest SOD (30.40 %) but low RSA (1.46 %) activities. Compound **3** (R = NHCH_2_-3-pyridiyl) as 3-pyridyl analog of compound **2** exerted lower SOD (25.22 %) and no RSA activities. As phenyl (C_6_H_5_) analog, compound **1** (R = NHC_6_H_5_) showed lower activities of SOD (27.26 %) and RSA (0.42 %) compared with compound **2**. Furan compound **4** (R = NHCH_2_-2-tetrahydrofuranyl) displayed the highest RSA (1.82 %).

The higher SOD activity of **2** could be due to its positive effect of descriptors with higher values of RDF120m (1.472) and Mor27u (-0.054) compared with compound **1 **showing lower values of RDF120m (0.000) and Mor27u (-0.070).

For RSA activity, **2** had higher RDF120m (1.472) and G2u (0.177) compared with **1** (RDF120m = 0.000, G2u = 0.175). It should be noted that RDF120m involved in both RSA and SOD activities making **2**>**1**. This high RDF120m value may be resulted from ring N-atom of 2-pyridyl ring of compound **2** compared with phenyl ring of compound **1**.

In case of 3-pyridyl ring, **3** (R = NHCH_2_-3-pyridyl) displayed lower SOD activity with lower RDF120m (0.240) and Mor27u (-0.091) compared with compound **2**. This might be due to an isomeric effect of pyridine ring, in which higher atomic polarizabilities (**2**, BELp5 = 1.167, **3**, BELp5 = 1.161) but lower 3^rd^ component symmetry (**2**, G3m = 0.170, **3**, G3m = 0.184) were noted for **2**. The R group of ring containing oxygen atom (**4**) displayed the highest RSA among group A. This could be due to its highest 2^nd^ component symmetry (G2u = 0.187) but the lowest van der Waals volume (H1v = 1.034) (Table 4(Tab. 4)).

Group B compounds **13**-**16** bearing longer ethylene linker between amino and phenyl/pyridyl rings, compound **15** gave the highest SOD (38.54 %) and RSA (4.62 %) activities, and higher activities than 2-pyridyl analog (**2**) with shorter CH_2 _linker.

The QSAR results showed that SOD activity of **15** (R with longer CH_2_CH_2_ linker) had the higher values of RDF120m (2.433) and Mor27u (0.082) than **2** containing shorter CH_2 _linker (RDF120m = 1.472, Mor27u = -0.054). This indicated the effect of higher mass RDF120m of **15** compared with **2**. Similar results were noted for RSA (**15**>**2**), in which **15 **had higher RDF120m = 2.433 and G2u = 0.191 compared with **2** (RDF120m = 1.472, G2u = 0.177).

Similar effect was noted for longer CH_2_CH_2_ linker compound **13** (SOD = 30.22 %, RSA = 4.36 %) compared with shorter linker compound **1** (SOD = 27.26 %, RSA = 0.42 %). This could be due to the higher RDF120m (0.205) and Mor27u (-0.006) of **13** in SOD activity compared with **1** (RDF120m = 0.000, Mor27u = -0.070). The similar results of descriptors were seen in RSA of compound **13** (RDF120m = 0.205, G2u = 0.209) and compound **1** (RDF120m = 0.000, G2u = 0.175), in which **13**>**1**.

3,4-Dimethoxyphenyl analog (**14**) showed higher SOD (32.24 %) activity, but lower RSA (0.82 %) compared with phenyl ring compound **13**. The QSAR result (SOD) of **14** showed higher values of RDF120m (2.737), BELp5 (1.393) and BELm8 (1.082) compared with **13** (RDF120m = 0.205, BELp5 = 1.296), BELm8= 1.000). On the other hand, compound **13** with higher RSA displayed high G2u (0.209), low Lop (1.055) whereas low G2u (0.177), high Lop (1.209) were noted for compound **14**. High polarizabilities BELp5 of **14** could be due to diOMe groups that enhance the molecule to scavenge superoxide in exerting the SOD activity. High value of G2u symmetry involved in RSA, thus, symmetrical compound **13** (without diOMe) displayed better RSA compared with compound **14**.

However, 2-pyridyl compound **15** exerted higher SOD (38.54 %) and RSA (4.62 %) compared with the phenyl analog **13**. This might be due to pyridine **15** with N-ring atom displayed SOD activity with higher RDF120m (2.433) compared with **13** (RDF120m = 0.205). Similar results were noted for RSA of **15** (RDF120m = 2.433) compared with **13** (RDF120m = 0.205).

In case of diamino ethane compound **16** (R = NHCH_2_CH_2_N(CH_2_CH_3_)_2_), relatively high SOD activity (32.85 %) was noted but the RSA was diminished. Among the investigated compounds, the highest BELp5 (1.422), but the lowest R7u (0.018) were observed for **16**. Such high polarizabilities might be due to the effect of polar diamino moieties.

Compounds in group D (**10**-**12**) with ring directly linked to the amino group, all displayed RSA and SOD activities. Cyclopentylamino compound (**11**) showed the highest SOD (31.74 %) but the lowest RSA (0.41 %), whereas cyclohexyl analog (**10**) exhibited higher RSA (1.29 %) activity. The highest Mor27u (0.158) and the highest BELp5 (1.334) were annotated for the SOD of **11** (R = NHcyclopentyl) compared with **10**, (R = NHcyclohexyl, Mor27u = 0.053, BELp5 = 1.236). This could be the ring size effect, as the smaller ring (**11**) had higher polarizabilities (BELp5) and Mor27u compared with larger ring (**10**). Compound **10** with higher RSA had high RDF120m (0.148), RDF045m (10.877), but low Lop (1.113) compared with compound **11** having low RDF120m = 0.054, RDF045m = 9.653, but high Lop = 1.144. The higher RSA might be due to high values of mass descriptors of **10** resulting from larger cyclohexyl ring.

Interestingly, in group D compound **12** (R = NHphenyl) had the highest RSA (1.91 %) with the lowest N-067 = 0, but the lowest SOD activity (29.35 %) with the lowest Mor27u (-0.054), BELp5 (0.998) and BELm8 (0.721). This could be due to the ability of phenyl ring that can stabilize the radical species (N^•^) producing by R group (NH-phenyl) and eventually form resonance stabilized phenyl radical.

Group C compounds (**5**-**9**) containing N-heterocyclic ring (R), the results showed that tetrahydroisoquinoline (**9**) exhibited the highest SOD (29.94 %) and RSA (3.70 %) activities. The highest SOD activity of **9** showed the highest Mor27u (-0.076) but the lowest R7u+ (0.020) among the group (**6**, **8** and **9**). Compound **9** with the highest RSA displayed the highest G2u (0.194) but the lowest H1v (1.288) among the group (**6**-**9**).

Compound **5** (R = *N*-methylpiperazine) was shown to be an inactive antioxidant. The improved activity was noted for *N*-benzylpiperazine **6** with RSA (2.73 %) and SOD (29.58 %). N-CH_3_ group of compound **5** was converted to NCH_2_C_6_H_5_ giving rise to compound **6** with improved RSA (2.73 %) and SOD (29.58 %) activities. The SOD activity of compound **6** had the highest RDF120m (0.681), BELp5 (1.409), BELm8 (1.082) but the lowest R7u+ (0.17) among the group (**6**, **8**-**9**). The RSA of **6** showed the lowest Lop (0.958) among the group (**6**-**9**).

In addition, morpholine compound **8** showed both RSA (1.11 %) and SOD (25.80 %) activities. Piperazine bearing bis-sulfonylphenylacetamido moiety (**7**) exhibited RSA (2.17 %), but no SOD activity. Compound **8** showed the lowest value of G3m (0.163) in SOD activity and the lowest Hlv value (1.135) in RSA among the group (**6**-**9**). Compound **7** displayed only the RSA with the highest RDF120m (0.875) and RDF045m (15.337)

To improve the antioxidant activities, the strongest parent antioxidant (**15**) was rationally designed by modifying its 2-pyridyl moiety as 3- and 4-pyridyl rings. These pyridyls were substituted by electron donating (EDG)/withdrawing (EWG) groups (i.e., OH, OCH_3_, NH_2_, SH, CN, NO_2_, COH and COOH) at various positions on the rings to give a series of new sulfonamides with predicted activities using the constructed QSAR equations (Eqs. 4-5) (Table S3, Supplementary information). The obtained values of descriptors for RSA and SOD activities are summarized (Tables S4 and S5, Supplementary information).

It was found that most of the newly designed sulfonamide derivatives displayed the improved RSA and SOD activities (Table S3, Supplementary information) with better molecular descriptor values than the parent compound **15** (Tables 4(Tab. 4) and 5(Tab. 5)). Top five antioxidant sulfonamides were selected from the 80 rationally designed compounds (Table S3, Supplementary information) including compounds **15-30** (*o*-NO_2_) > **15-22** (*m*-NO_2_) > **15-5** (*m*-CN) > **15-28** (*o*-SH) > **15-49** (*m*-OH) with RSA as shown in Table 6(Tab. 6), and compounds with SOD activity (Table 7(Tab. 7)); **15-14 **(*p*-NO2) > **15-22 **(*m*-NO_2_) > **15-47** (*p*-COH) > **15-30** (*o*-NO2) > **15-49** (*m*-OH). 

**Figure 3:**
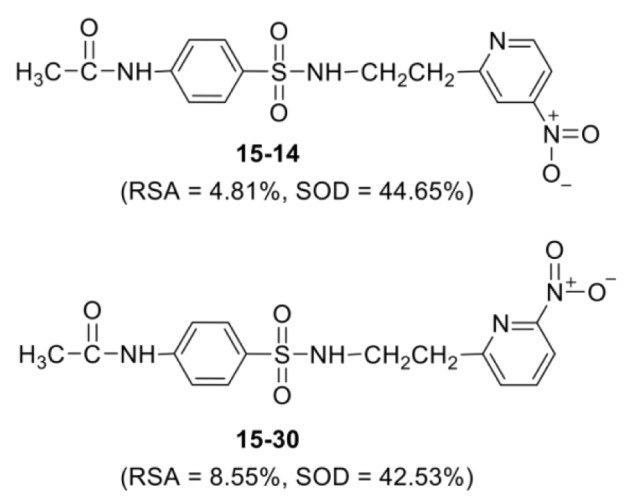
New designed nitrosulfonamides with RSA and SOD activities

**Figure 4:**
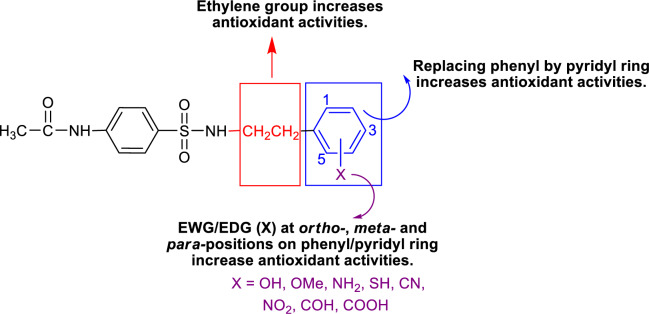
SAR analysis of sulfonamide derivatives

Most of the active compounds are 2-pyridyl containing electron withdrawing groups (i.e., NO_2_, CN and CHO) at *ortho*/*para* position on ring N-atom. The most active RSA **15-30** (*o*-NO_2_) had high values of G2u (0.227), RDF120m (4.311) and RDF045m (11.257), but low H1v (1.138) compared with the parent compound **15** (G2u = 0.191, RDF120m = 2.433, RDF045m = 8.415, H1v = 1.222). As *p*-NO_2_ analog (**15-14**), it displayed the highest SOD activity with high Mor27u (0.190), BELm8 (0.993), BELp5 (1.475), RDF120m (3.787) but low R7u (0.023), and G3m (0.157) compared with the compound **15** (Mor27u = 0.082, BELm8 = 0.926, BELp5 = 1.296, RDF120m = 2.433, R7u = 0.026, G3m = 0.159). The highest SOD activity of **15-14** might be due to high polarizabilities (BELp5 = 1.475) resulting from ionic character of NO_2_ group (Figure 3(Fig. 3)) and its *p*-effect on 2-pyridyl ring, which could facilitate superoxide scavenging activity. In addition, *o*-NO_2_ (**15-30**) and *m*-NO_2_ (**15-22**) analogs are also the top five SOD activity (Table 7(Tab. 7)). Particularly, both improved RSA and SOD activities are seen for new designed compounds **15-22** and **15-30. **The structure-activity relationship analysis is summarized in Figure 4(Fig. 4).

### Drug likeness properties

The investigated sulfonamides were determined for drug likeness characters based on the Lipinski rule of five with the molecular features composed of (1) MW < 500 Da, (2) LogP < 5, (3) nHDon < 5, and (4) nHAcc < 10 using Dragon software. The description of descriptors are as followed: molecular weight (MW) generally represents the molecular size of the compounds, LogP is a parameter to determine molecular hydrophobicity or lipophilicity character of the 1-octanol/water partition coefficient, nHDon and nHAcc were the number of hydrogen bond donors (OH and NH groups) and acceptors (N and O), respectively as well as hydrogen bonding capacity in the molecules (Nantasenamat et al., 2013[23]). It was found that all sulfonamides exhibited drug likeness properties (Table S6, Supplementary information). In addition, such drug likeness properties were also noted for the rational design of 80 new sulfonamides (Table S7, Supplementary information).

## Conclusion

Acetamidosulfonamide derivatives were synthesized and evaluated for their antioxidant and antimicrobial activities. Most sulfonamides exhibited antioxidant activities, particularly, compound **15** exhibited the highest RSA and SOD activities, but all sulfonamides revealed to be inactive antimicrobials. The SAR indicated that the compound bearing 2C chain length with terminal 2-pyridyl ring (**15**) displayed higher antioxidant activities compared with the corresponding compound with 1C chain (**2**). Furthermore, the constructed QSAR models displayed important descriptors related with the antioxidant activities (i.e., G2u, Lop, RDF120m, N-067, H1v and RDF045m for RSA, while RDF120m, Mor27u, R7u+, BELp5, G3m and BELm8 for SOD activity. This result led to *in silico* rational design of 80 new sulfonamide derivatives with electron donating and electron withdrawing groups together with the calculated predicting antioxidant activities using the prototype compound **15**. The top five newly designed sulfonamides i.e., nitro compound **15-30** showed the improved antioxidant RSA and SOD activities. In addition, these sulfonamides possessed drug likeness properties. This finding revealed the benefit of constructed QSAR models to insight into the rational design of new antioxidants to be further developed for medicinal applications.

## Notes

Apilak Worachartcheewan and Supaluk Prachayasittikul (Center of Data Mining and Biomedical Informatics, Faculty of Medical Technology, Mahidol University, Bangkok 10700, Thailand; E-mail: supaluk@g.swu.ac.th) contributed equally as corresponding author.

## Declaration

### Supplementary information

Supplementary information is available on the EXCLI Journal website.

### Conflict of interest

The authors declare that they have no conflict of interest.

### Acknowledgments

AW gratefully acknowledges the research grant supported by the Thailand Research Fund (Grant No. MRG6180053) and the annual budget grant from Mahidol University (B.E. 2562-2563).

## Supplementary Material

Supplementary information

## Figures and Tables

**Table 1:**
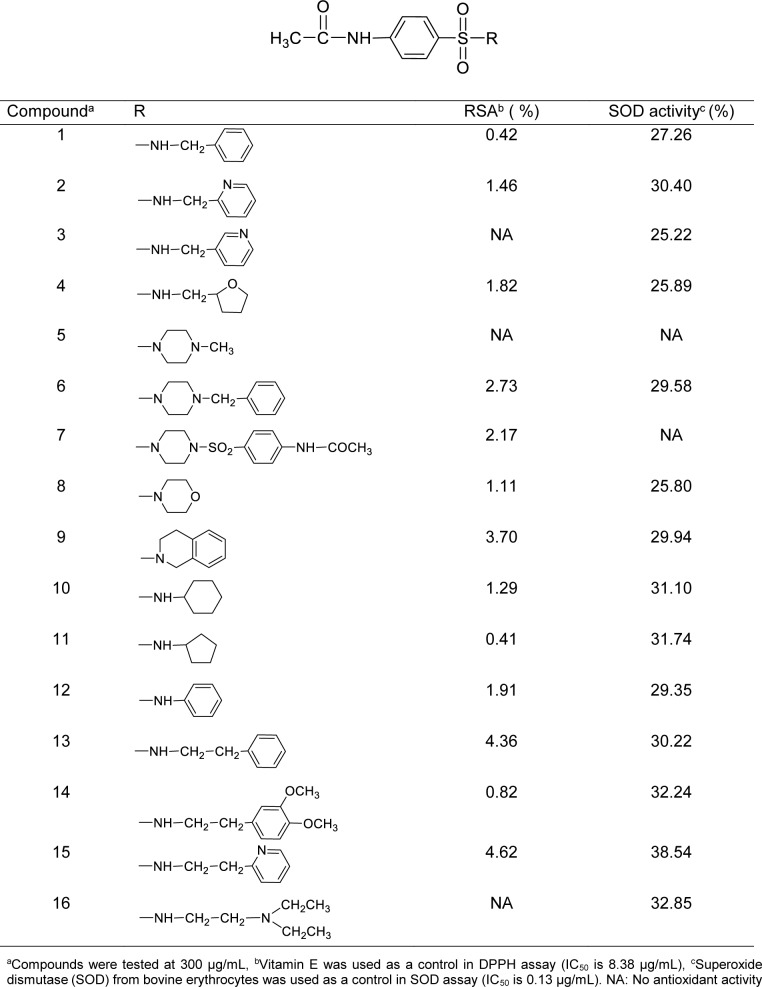
Antioxidant activities (RSA and SOD) of sulfonamide derivatives

**Table 2:**
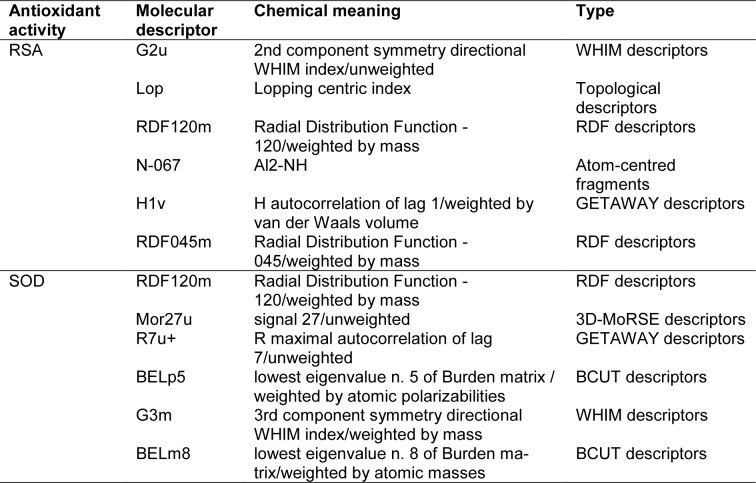
Definition and types of significant molecular descriptors for QSAR construction

**Table 3:**
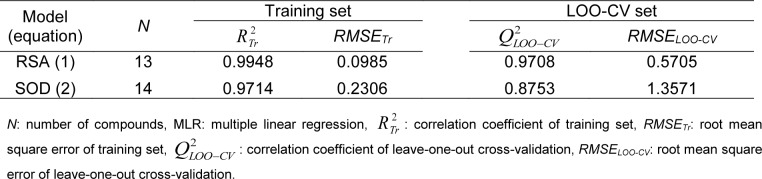
Statistical results of predictive QSAR models using MLR method

**Table 4:**
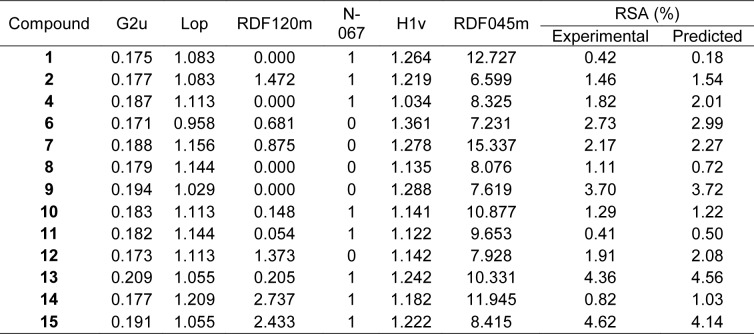
Values of molecular descriptors and predicted RSA of sulfonamides

**Table 5:**
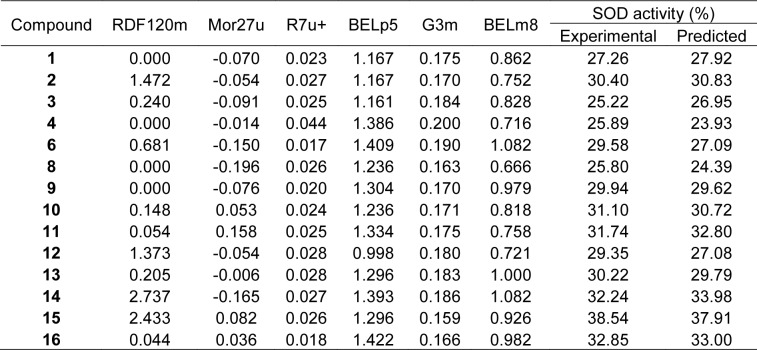
Values of molecular descriptors and predicted SOD activity of sulfonamides

**Table 6:**
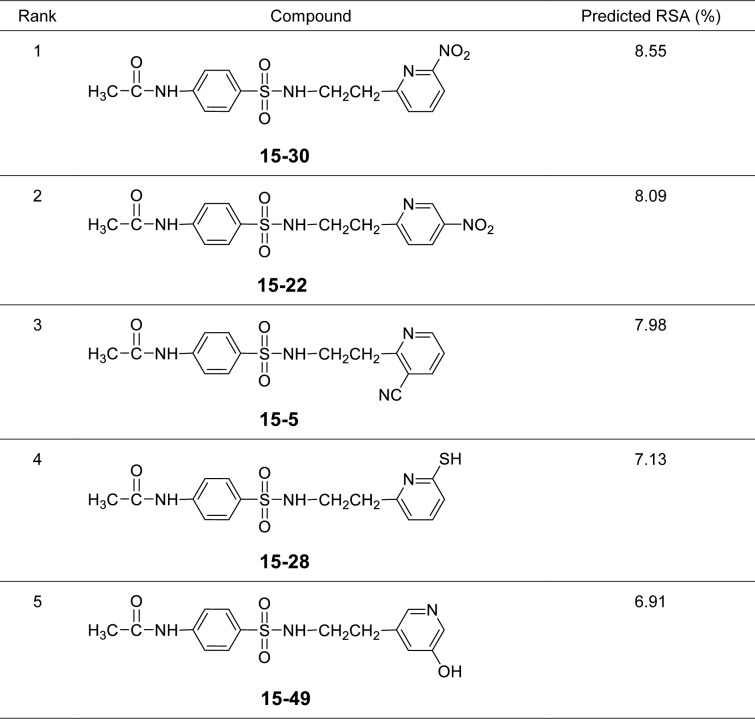
Top five of predicted new sulfonamides with RSA

**Table 7:**
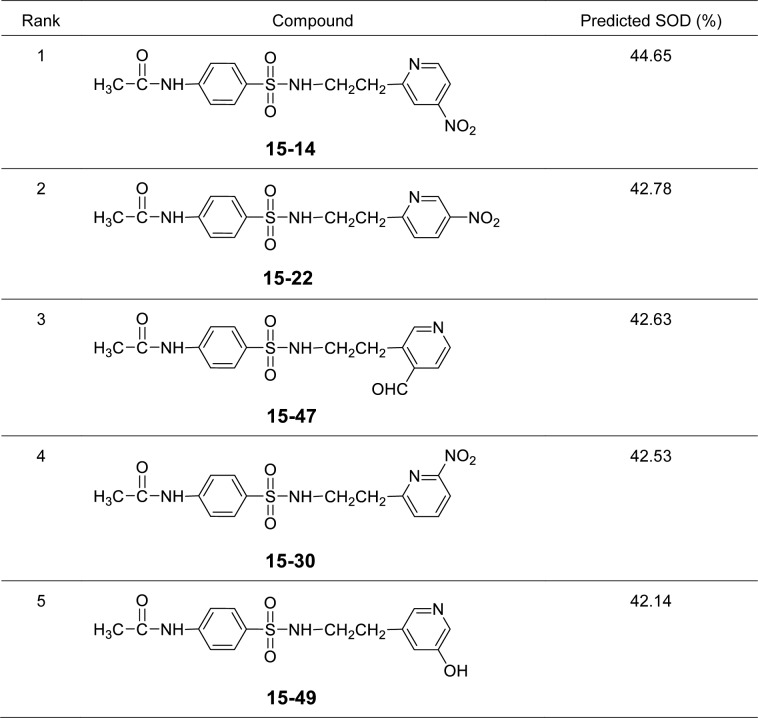
Top five of predicted new sulfonamides with SOD activity
