# Supplementary material for: Synthesis of acetamidosulfonamide derivatives with antioxidative and QSAR studies
Source: EXCLI J. 2022 Feb 1;21:360–79. doi: 10.17179/excli2021-4590 (PMC9618713; doi:10.17179/excli2021-4590)
Supplement: Supplementary information [file EXCLI-21-360-s-001.pdf]

EXCLI Journal 2022;21:360-379 – ISSN 1611-2156
Received: December 09, 2021, accepted: January 11, 2022, published: February 01, 2022

Supplementary information to:

Original article:

SYNTHESIS OF ACETAMIDOSULFONAMIDE DERIVATIVES WITH ANTIOXIDATIVE AND QSAR STUDIES

Apilak Worachartcheewan[1*], Somchai Pisutjaroenpong[2], Ratchanok Pingaew[3], Supaluk Prachayasittikul[4*], Suphakit Siriwong[1], Somsak Ruchirawat[2,5,6], Virapong Prachayasittikul[7]

[1] Department of Community Medical Technology, Faculty of Medical Technology, Mahidol University, Bangkok 10700, Thailand
[2] Laboratory of Medicinal Chemistry, Chulabhorn Research Institute, Bangkok 10210, Thailand
[3] Department of Chemistry, Faculty of Science, Srinakharinwirot University, Bangkok 10110, Thailand
[4] Center of Data Mining and Biomedical Informatics, Faculty of Medical Technology, Mahidol University, Bangkok 10700, Thailand
[5] Program in Chemical Science, Chulabhorn Graduate Institute, Bangkok 10210, Thailand
[6] Center of Excellence on Environmental Health and Toxicology, Commission on Higher Education (CHE), Ministry of Education, Thailand
[7] Department of Clinical Microbiology and Applied Technology, Faculty of Medical Technology, Mahidol University, Bangkok 10700, Thailand

* **Corresponding authors:** Assistant Professor Dr. Apilak Worachartcheewan, Department of Community Medical Technology, Faculty of Medical Technology, Mahidol University, Bangkok 10700, Thailand, Phone: (662) 441-4376; Fax: (662) 441-4380;
E-mail: apilak.woa@mahidol.ac.th
Professor Dr. Supaluk Prachayasittikul, Center of Data Mining and Biomedical Informatics, Faculty of Medical Technology, Mahidol University, Bangkok 10700, Thailand; E-mail: supaluk@g.swu.ac.th

https://dx.doi.org/10.17179/excli2020-4590

This is an Open Access article distributed under the terms of the Creative Commons Attribution License (http://creativecommons.org/licenses/by/4.0/).

EXCLI Journal 2022;21:360-379 – ISSN 1611-2156
Received: December 09, 2021, accepted: January 11, 2022, published: February 01, 2022

Table S1: Intercorrelation matrix of significant descriptors for predicted RSA

	G2u	Lop	RDF120m	N-067	H1v	RDF045m
G2u	1.000					
Lop	-0.128	1.000				
RDF120m	-0.195	0.222	1.000			
N-067	0.201	0.213	0.155	1.000		
H1v	0.038	-0.61	0.102	-0.363	1.000	
RDF045m	0.131	0.507	0.013	0.124	0.122	1.000

Table S2: Intercorrelation matrix of significant descriptors for predicted SOD activity

	RDF120m	Mor27u	R7u+	BELp5	G3m	BELm8
RDF120m	1.000					
Mor27u	-0.119	1.000				
R7u+	0.055	0.089	1.000			
BELp5	-0.042	0.1	-0.063	1.000		
G3m	-0.079	-0.244	0.49	0.186	1.000	
BELm8	0.287	-0.151	-0.51	0.565	0.117	1.000

EXCLI Journal 2022;21:360-379 – ISSN 1611-2156
Received: December 09, 2021, accepted: January 11, 2022, published: February 01, 2022

Table S3: New 80 rationally designed sulfonamide derivatives with predicted antioxidant activities

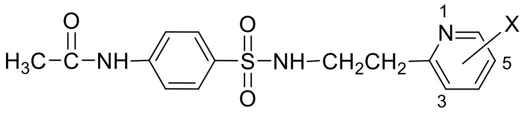

15-(1-32)

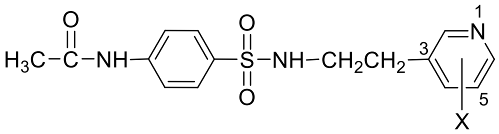

15-(33-64)

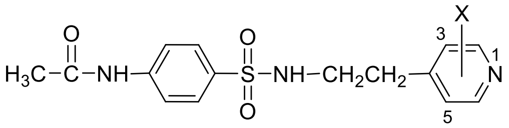

15-(65-80)

Compound	X	Predicted activity (%)	
		RSA	SOD
**15-1**	3-OH	0.34	25.13
**15-2**	3-OCH_3	NA	29.63
**15-3**	3-NH_2	4.40	28.16
**15-4**	3-SH	6.42	42.14
**15-5**	3-CN	7.98	34.20
**15-6**	3-NO_2	2.17	28.66
**15-7**	3-COH	0.15	32.78
**15-8**	3-COOH	NA	30.57
**15-9**	4-OH	4.88	36.45
**15-10**	4-OCH_3	3.73	27.62
**15-11**	4-NH_2	4.48	27.26
**15-12**	4-SH	4.43	40.00
**15-13**	4-CN	1.89	34.18
**15-14**	4-NO_2	4.81	44.65
**15-15**	4-COH	5.67	37.88
**15-16**	4-COOH	3.06	29.96
**15-17**	5-OH	1.82	32.13
**15-18**	5-OCH_3	5.31	33.69
**15-19**	5-NH_2	2.14	26.09
**15-20**	5-SH	2.90	31.57
**15-21**	5-CN	3.98	31.61
**15-22**	5-NO_2	8.09	42.78
**15-23**	5-COH	6.67	38.26
**15-24**	5-COOH	0.78	26.99

EXCLI Journal 2022;21:360-379 – ISSN 1611-2156
Received: December 09, 2021, accepted: January 11, 2022, published: February 01, 2022

Compound	X	Predicted activity (%)	
		RSA	SOD
**15-25**	6-OH	NA	31.33
**15-26**	6-OCH_3	4.74	37.09
**15-27**	6-NH_2	NA	30.13
**15-28**	6-SH	7.13	40.68
**15-29**	6-CN	4.00	36.01
**15-30**	6-NO_2	8.55	42.53
**15-31**	6-COH	5.46	35.47
**15-32**	6-COOH	NA	35.19
**15-33**	2-OH	NA	30.61
**15-34**	2-OCH_3	0.07	27.89
**15-35**	2-NH_2	NA	34.87
**15-36**	2-SH	5.98	35.16
**15-37**	2-CN	NA	29.94
**15-38**	2-NO_2	NA	30.26
**15-39**	2-COH	2.38	37.82
**15-40**	2-COOH	4.21	36.56
**15-41**	4-OH	2.95	27.74
**15-42**	4-OCH_3	NA	28.11
**15-43**	4-NH_2	3.72	29.99
**15-44**	4-SH	1.29	31.14
**15-45**	4-CN	NA	28.36
**15-46**	4-NO_2	NA	31.25
**15-47**	4-COH	4.84	42.63
**15-48**	4-COOH	0.94	30.08
**15-49**	5-OH	6.91	29.12
**15-50**	5-OCH_3	1.13	25.71
**15-51**	5-NH_2	3.04	31.41
**15-52**	5-SH	NA	30.02
**15-53**	5-CN	4.10	39.54
**15-54**	5-NO_2	NA	30.40
**15-55**	5-COH	3.07	30.00
**15-56**	5-COOH	2.18	29.15
**15-57**	6-OH	NA	27.84
**15-58**	6-OCH_3	0.67	34.44
**15-59**	6-NH_2	3.68	33.91
**15-60**	6-SH	6.52	29.92

EXCLI Journal 2022;21:360-379 – ISSN 1611-2156
Received: December 09, 2021, accepted: January 11, 2022, published: February 01, 2022

Compound	X	Predicted activity (%)	
		RSA	SOD
**15-61**	6-CN	4.08	33.47
**15-62**	6-NO_2	1.37	33.53
**15-63**	6-COH	4.10	29.20
**15-64**	6-COOH	6.45	39.06
**15-65**	2-OH	2.85	37.34
**15-66**	2-OCH_3	2.72	38.99
**15-67**	2-NH_2	1.66	30.29
**15-68**	2-SH	4.05	32.71
**15-69**	2-CN	0.98	32.05
**15-70**	2-NO_2	1.89	23.00
**15-71**	2-COH	NA	30.81
**15-72**	2-COOH	NA	22.60
**15-73**	3-OH	0.51	26.60
**15-74**	3-OCH_3	0.77	28.05
**15-75**	3-NH_2	5.26	37.45
**15-76**	3-SH	1.92	36.16
**15-77**	3-CN	0.56	32.38
**15-78**	3-NO_2	NA	33.55
**15-79**	3-COH	NA	27.87
**15-80**	3-COOH	NA	25.46

NA: no activity

EXCLI Journal 2022;21:360-379 – ISSN 1611-2156
Received: December 09, 2021, accepted: January 11, 2022, published: February 01, 2022

Table S4: Molecular descriptor values of 80 new sulfonamide derivatives for predicted RSA

Compound	G2u	Lop	RDF120m	N-067	H1v	RDF045m
**15-1**	0.171	1.069	0.006	1	1.232	12.530
**15-2**	0.178	1.152	1.003	1	1.258	5.825
**15-3**	0.194	1.069	1.127	1	1.072	10.676
**15-4**	0.183	1.069	4.373	1	1.205	23.425
**15-5**	0.238	1.152	2.530	1	1.21	12.781
**15-6**	0.194	1.157	1.461	1	1.246	11.962
**15-7**	0.170	1.152	1.712	1	1.217	11.701
**15-8**	0.180	1.157	0.071	1	1.252	8.409
**15-9**	0.183	1.069	2.860	1	1.052	10.887
**15-10**	0.208	1.152	1.051	1	1.142	9.558
**15-11**	0.200	1.069	0.734	1	1.106	10.472
**15-12**	0.177	1.069	3.676	1	1.165	12.091
**15-13**	0.183	1.152	3.065	1	1.261	6.005
**15-14**	0.194	1.157	3.787	1	1.104	10.361
**15-15**	0.194	1.152	4.409	1	1.065	10.524
**15-16**	0.192	1.157	2.466	1	1.146	8.280
**15-17**	0.171	1.069	1.679	1	1.169	9.077
**15-18**	0.201	1.152	3.23	1	1.079	10.525
**15-19**	0.194	1.069	0.008	1	1.235	6.278
**15-20**	0.183	1.069	1.653	1	1.225	11.092
**15-21**	0.209	1.152	1.925	1	1.208	7.003
**15-22**	0.220	1.157	4.543	1	1.116	10.565
**15-23**	0.220	1.152	3.904	1	1.231	6.905
**15-24**	0.180	1.157	1.580	1	1.175	6.837
**15-25**	0.158	1.069	0.094	1	1.225	9.962
**15-26**	0.195	1.152	3.159	1	1.060	10.587
**15-27**	0.157	1.069	0.000	1	1.265	8.127
**15-28**	0.183	1.069	5.589	1	1.109	13.621
**15-29**	0.209	1.152	2.204	1	1.286	9.511
**15-30**	0.227	1.157	4.311	1	1.138	11.257
**15-31**	0.194	1.152	4.492	1	1.118	10.727
**15-32**	0.180	1.157	0.000	1	1.325	12.768
**15-33**	0.171	1.069	0.000	1	1.322	7.291
**15-34**	0.190	1.152	0.000	1	1.271	7.403
**15-35**	0.157	1.069	0.000	1	1.300	6.925
**15-36**	0.196	1.069	2.926	1	1.160	14.469
**15-37**	0.171	1.152	1.537	1	1.241	9.703

EXCLI Journal 2022;21:360-379 – ISSN 1611-2156
Received: December 09, 2021, accepted: January 11, 2022, published: February 01, 2022

Compound	G2u	Lop	RDF120m	N-067	H1v	RDF045m
**15-38**	0.182	1.157	0.000	1	1.328	12.779
**15-39**	0.182	1.152	2.132	1	1.120	12.751
**15-40**	0.204	1.157	1.682	1	1.123	13.451
**15-41**	0.196	1.069	0.007	1	1.195	10.595
**15-42**	0.168	1.152	0.315	1	1.208	8.889
**15-43**	0.206	1.069	0.000	1	1.250	11.202
**15-44**	0.183	1.069	0.000	1	1.325	15.224
**15-45**	0.171	1.152	0.985	1	1.360	5.638
**15-46**	0.170	1.157	0.410	1	1.252	11.345
**15-47**	0.206	1.152	2.297	1	1.168	14.026
**15-48**	0.180	1.157	1.564	1	1.228	12.131
**15-49**	0.223	1.069	1.464	1	1.196	9.969
**15-50**	0.190	1.152	0.078	1	1.195	13.887
**15-51**	0.206	1.069	0.021	1	1.300	6.500
**15-52**	0.171	1.069	0.361	1	1.358	9.159
**15-53**	0.189	1.152	4.753	1	1.276	8.612
**15-54**	0.182	1.157	0.399	1	1.244	10.921
**15-55**	0.194	1.152	1.753	1	1.109	9.473
**15-56**	0.204	1.157	0.404	1	1.173	5.967
**15-57**	0.158	1.069	0.732	1	1.206	9.888
**15-58**	0.178	1.152	1.872	1	1.207	6.109
**15-59**	0.170	1.069	3.647	1	1.134	10.121
**15-60**	0.223	1.069	0.653	1	1.174	11.919
**15-61**	0.202	1.152	2.841	1	1.234	9.087
**15-62**	0.194	1.157	0.310	1	1.205	11.324
**15-63**	0.206	1.152	1.698	1	1.145	9.982
**15-64**	0.204	1.157	4.220	1	1.047	9.390
**15-65**	0.171	1.069	2.885	1	1.179	9.783
**15-66**	0.190	1.152	2.001	1	1.140	9.825
**15-67**	0.182	1.069	0.171	1	1.182	9.812
**15-68**	0.196	1.069	0.855	1	1.178	13.680
**15-69**	0.183	1.152	1.659	1	1.299	11.695
**15-70**	0.200	1.157	0.000	1	1.275	17.763
**15-71**	0.170	1.152	0.000	1	1.285	16.196
**15-72**	0.156	1.157	0.000	1	1.276	18.435
**15-73**	0.171	1.069	0.000	1	1.271	16.950
**15-74**	0.19	1.152	0.000	1	1.302	17.310
**15-75**	0.200	1.069	1.675	1	1.147	12.829

EXCLI Journal 2022;21:360-379 – ISSN 1611-2156
Received: December 09, 2021, accepted: January 11, 2022, published: February 01, 2022

Compound	G2u	Lop	RDF120m	N-067	H1v	RDF045m
**15-76**	0.177	1.069	1.529	1	1.284	12.010
**15-77**	0.196	1.152	0.000	1	1.353	11.192
**15-78**	0.170	1.157	0.000	1	1.279	27.558
**15-79**	0.157	1.152	0.000	1	1.290	16.341
**15-80**	0.169	1.157	0.000	1	1.286	18.067

EXCLI Journal 2022;21:360-379 – ISSN 1611-2156
Received: December 09, 2021, accepted: January 11, 2022, published: February 01, 2022

Table S5: Molecular descriptor values of 80 new sulfonamide derivatives for predicted SOD activity

Compound	RDF120m	Mor27u	R7u+	BELp5	G3m	BELm8
**15-1**	0.006	-0.171	0.035	1.296	0.189	0.917
**15-2**	1.003	-0.286	0.019	1.374	0.156	0.970
**15-3**	1.127	-0.047	0.026	1.296	0.206	0.946
**15-4**	4.373	0.088	0.028	1.296	0.158	0.885
**15-5**	2.530	-0.153	0.035	1.296	0.158	0.941
**15-6**	1.461	-0.287	0.023	1.508	0.176	0.944
**15-7**	1.712	0.098	0.024	1.297	0.200	0.947
**15-8**	0.071	-0.109	0.028	1.331	0.156	0.945
**15-9**	2.860	0.011	0.024	1.303	0.171	0.909
**15-10**	1.051	-0.209	0.026	1.381	0.190	1.012
**15-11**	0.734	-0.083	0.020	1.317	0.206	0.999
**15-12**	3.676	0.051	0.023	1.293	0.158	0.873
**15-13**	3.065	-0.095	0.020	1.317	0.183	0.982
**15-14**	3.787	0.190	0.023	1.475	0.157	0.993
**15-15**	4.409	0.016	0.025	1.329	0.194	1.001
**15-16**	2.466	-0.287	0.021	1.359	0.18	0.994
**15-17**	1.679	-0.097	0.028	1.309	0.171	0.926
**15-18**	3.230	-0.247	0.021	1.390	0.168	0.988
**15-19**	0.008	-0.187	0.019	1.332	0.182	0.930
**15-20**	1.653	-0.199	0.024	1.289	0.158	0.925
**15-21**	1.925	-0.053	0.024	1.331	0.189	0.928
**15-22**	4.543	0.112	0.022	1.469	0.170	0.929
**15-23**	3.904	-0.029	0.024	1.346	0.170	0.930
**15-24**	1.580	-0.257	0.021	1.374	0.192	0.929
**15-25**	0.094	-0.001	0.022	1.303	0.165	0.909
**15-26**	3.159	0.005	0.021	1.388	0.178	0.979
**15-27**	0.000	-0.129	0.018	1.320	0.157	0.959
**15-28**	5.589	0.005	0.025	1.293	0.177	0.850
**15-29**	2.204	-0.038	0.026	1.319	0.158	0.952
**15-30**	4.311	0.078	0.028	1.485	0.164	0.956
**15-31**	4.492	0.048	0.025	1.334	0.220	0.960
**15-32**	0.000	0.128	0.024	1.368	0.156	0.957
**15-33**	0.000	-0.060	0.018	1.292	0.158	0.880
**15-34**	0.000	-0.201	0.023	1.380	0.168	0.978
**15-35**	0.000	0.130	0.018	1.292	0.157	0.960
**15-36**	2.926	-0.020	0.028	1.292	0.171	0.809
**15-37**	1.537	-0.137	0.022	1.292	0.183	0.951

EXCLI Journal 2022;21:360-379 – ISSN 1611-2156
Received: December 09, 2021, accepted: January 11, 2022, published: February 01, 2022

Compound	RDF120m	Mor27u	R7u+	BELp5	G3m	BELm8
**15-38**	0.000	-0.087	0.022	1.512	0.170	0.956
**15-39**	2.132	0.063	0.026	1.293	0.157	0.961
**15-40**	1.682	0.028	0.026	1.341	0.156	0.957
**15-41**	0.007	-0.059	0.029	1.292	0.183	0.886
**15-42**	0.315	-0.176	0.020	1.370	0.178	1.010
**15-43**	0.000	0.001	0.020	1.292	0.182	1.009
**15-44**	0.000	0.093	0.020	1.292	0.177	0.854
**15-45**	0.985	-0.185	0.018	1.292	0.183	1.003
**15-46**	0.410	-0.167	0.031	1.500	0.157	1.009
**15-47**	2.297	0.280	0.023	1.293	0.157	1.009
**15-48**	1.564	-0.216	0.022	1.324	0.174	1.009
**15-49**	1.464	-0.077	0.023	1.301	0.196	0.900
**15-50**	0.078	-0.265	0.025	1.385	0.178	0.976
**15-51**	0.021	0.023	0.017	1.320	0.170	0.935
**15-52**	0.361	-0.128	0.017	1.287	0.158	0.876
**15-53**	4.753	-0.112	0.028	1.319	0.158	0.924
**15-54**	0.399	0.041	0.025	1.476	0.194	0.930
**15-55**	1.753	-0.095	0.024	1.334	0.194	0.937
**15-56**	0.404	-0.119	0.025	1.365	0.174	0.932
**15-57**	0.732	-0.216	0.022	1.306	0.171	0.897
**15-58**	1.872	-0.065	0.020	1.393	0.168	0.994
**15-59**	3.647	-0.028	0.025	1.331	0.206	0.964
**15-60**	0.653	-0.162	0.022	1.284	0.158	0.880
**15-61**	2.841	0.018	0.023	1.330	0.202	0.944
**15-62**	0.310	-0.010	0.027	1.473	0.157	0.955
**15-63**	1.698	-0.073	0.027	1.347	0.206	0.967
**15-64**	4.220	-0.045	0.024	1.377	0.169	0.958
**15-65**	2.885	-0.022	0.032	1.294	0.158	0.931
**15-66**	2.001	0.088	0.021	1.388	0.156	1.009
**15-67**	0.171	-0.051	0.031	1.314	0.170	0.960
**15-68**	0.855	-0.045	0.023	1.282	0.158	0.909
**15-69**	1.659	-0.105	0.035	1.313	0.171	0.952
**15-70**	0.000	-0.397	0.019	1.482	0.182	0.956
**15-71**	0.000	-0.092	0.024	1.331	0.157	0.961
**15-72**	0.000	-0.403	0.019	1.367	0.180	0.957
**15-73**	0.000	-0.208	0.030	1.286	0.171	0.937
**15-74**	0.000	-0.275	0.019	1.375	0.156	1.003
**15-75**	1.675	0.099	0.023	1.287	0.157	0.942

EXCLI Journal 2022;21:360-379 – ISSN 1611-2156
Received: December 09, 2021, accepted: January 11, 2022, published: February 01, 2022

Compound	RDF120m	Mor27u	R7u+	BELp5	G3m	BELm8
**15-76**	1.529	0.134	0.025	1.286	0.171	0.934
**15-77**	0.000	0.020	0.029	1.287	0.158	0.940
**15-78**	0.000	0.174	0.031	1.503	0.182	0.941
**15-79**	0.000	-0.224	0.033	1.287	0.157	0.943
**15-80**	0.000	-0.154	0.034	1.333	0.192	0.941

EXCLI Journal 2022;21:360-379 – ISSN 1611-2156
Received: December 09, 2021, accepted: January 11, 2022, published: February 01, 2022

Table S6: Lipinski rule of five of sulfonamide derivatives (**1**-**16**)

Compound	MW	nHDon	nHAcc	ALogP	Rule of five[a]
**1**	304.40	2	5	1.640	***0***
**2**	305.39	2	6	0.917	***0***
**3**	305.39	2	6	0.489	***0***
**4**	298.40	2	6	0.415	***0***
**5**	297.42	1	6	0.549	***0***
**6**	373.52	1	6	2.133	***0***
**7**	480.62	2	10	0.821	***0***
**8**	284.37	1	6	0.280	***0***
**9**	330.44	1	5	2.250	***0***
**10**	296.43	2	5	1.914	***0***
**11**	282.40	2	5	1.458	***0***
**12**	290.37	2	5	1.911	***0***
**13**	318.43	2	5	1.961	***0***
**14**	378.49	2	7	1.928	***0***
**15**	319.42	2	6	0.952	***0***
**16**	313.47	2	6	0.892	***0***

MW: molecular weight <500; nHDon: number of hydrogen bond donor <5; nHAcc: number of hydrogen bond acceptor; AlogP: partition coefficient
[a]Rule of five-number of Lipinsky rule violation

EXCLI Journal 2022;21:360-379 – ISSN 1611-2156
Received: December 09, 2021, accepted: January 11, 2022, published: February 01, 2022

Table S7: Lipinski rule of five of 80 new rationally designed sulfonamide derivatives

Compound	MW	nHDon	nHAcc	ALogP	Rule of five[a]
**15-1**	335.42	3	7	0.685	0
**15-2**	349.45	2	7	0.936	0
**15-3**	334.44	4	7	0.206	0
**15-4**	351.49	2	6	1.312	0
**15-5**	344.43	2	7	0.831	0
**15-6**	364.42	2	8	0.847	0
**15-7**	347.43	2	7	0.712	0
**15-8**	363.43	3	8	0.557	0
**15-9**	335.42	3	7	0.685	0
**15-10**	349.45	2	7	0.936	0
**15-11**	334.44	4	7	0.206	0
**15-12**	351.49	2	6	1.312	0
**15-13**	344.43	2	7	0.831	0
**15-14**	364.42	2	8	0.847	0
**15-15**	347.43	2	7	0.712	0
**15-16**	363.43	3	8	0.557	0
**15-17**	335.42	3	7	0.685	0
**15-18**	349.45	2	7	0.936	0
**15-19**	334.44	4	7	0.206	0
**15-20**	351.49	2	6	1.312	0
**15-21**	344.43	2	7	0.831	0
**15-22**	364.42	2	8	0.847	0
**15-23**	347.43	2	7	0.712	0
**15-24**	363.43	3	8	0.557	0
**15-25**	335.42	3	7	1.224	0
**15-26**	349.45	2	7	1.475	0
**15-27**	334.44	4	7	0.745	0
**15-28**	351.49	2	6	1.851	0
**15-29**	344.43	2	7	1.260	0
**15-30**	364.42	2	8	1.386	0
**15-31**	347.43	2	7	1.140	0
**15-32**	363.43	3	8	0.985	0
**15-33**	335.42	3	7	1.082	0
**15-34**	349.45	2	7	1.333	0
**15-35**	334.44	4	7	0.603	0
**15-36**	351.49	2	6	1.709	0
**15-37**	344.43	2	7	1.118	0

EXCLI Journal 2022;21:360-379 – ISSN 1611-2156
Received: December 09, 2021, accepted: January 11, 2022, published: February 01, 2022

Compound	MW	nHDon	nHAcc	ALogP	Rule of five[a]
**15-38**	364.42	2	8	1.244	0
**15-39**	347.43	2	7	0.998	0
**15-40**	363.43	3	8	0.843	0
**15-41**	335.42	3	7	0.543	0
**15-42**	349.45	2	7	0.794	0
**15-43**	334.44	4	7	0.063	0
**15-44**	351.49	2	6	1.170	0
**15-45**	344.43	2	7	0.689	0
**15-46**	364.42	2	8	0.704	0
**15-47**	347.43	2	7	0.569	0
**15-48**	363.43	3	8	0.414	0
**15-49**	335.42	3	7	0.543	0
**15-50**	349.45	2	7	0.794	0
**15-51**	334.44	4	7	0.063	0
**15-52**	351.49	2	6	1.170	0
**15-53**	344.43	2	7	0.689	0
**15-54**	364.42	2	8	0.704	0
**15-55**	347.43	2	7	0.569	0
**15-56**	363.43	3	8	0.414	0
**15-57**	335.42	3	7	1.082	0
**15-58**	349.45	2	7	1.333	0
**15-59**	334.44	4	7	0.603	0
**15-60**	351.49	2	6	1.709	0
**15-61**	344.43	2	7	1.118	0
**15-62**	364.42	2	8	1.244	0
**15-63**	347.43	2	7	0.998	0
**15-64**	363.43	3	8	0.843	0
**15-65**	335.42	3	7	1.082	0
**15-66**	349.45	2	7	1.333	0
**15-67**	334.44	4	7	0.603	0
**15-68**	351.49	2	6	1.709	0
**15-69**	344.43	2	7	1.118	0
**15-70**	364.42	2	8	1.244	0
**15-71**	347.43	2	7	0.998	0
**15-72**	363.43	3	8	0.843	0

EXCLI Journal 2022;21:360-379 – ISSN 1611-2156
Received: December 09, 2021, accepted: January 11, 2022, published: February 01, 2022

Compound	MW	nHDon	nHAcc	ALogP	Rule of five[a]
**15-73**	335.42	3	7	0.543	0
**15-74**	349.45	2	7	0.794	0
**15-75**	334.44	4	7	0.063	0
**15-76**	351.49	2	6	1.170	0
**15-77**	344.43	2	7	0.689	0
**15-78**	364.42	2	8	0.704	0
**15-79**	347.43	2	7	0.569	0
**15-80**	363.43	3	8	0.414	0

MW: molecular weight <500; nHDon: number of hydrogen bond donor <5; nHAcc: number of hydrogen bond acceptor; AlogP: partition coefficient
[a]Rule of five-number of Lipinsky rule violation
